# Salt marsh monitoring along the mid-Atlantic coast by Google Earth Engine enabled time series

**DOI:** 10.1371/journal.pone.0229605

**Published:** 2020-02-28

**Authors:** Anthony Daniel Campbell, Yeqiao Wang

**Affiliations:** Department of Natural Resources Science, University of Rhode Island Kingston, Kingston, Rhode Island, United States of America; University of Wisconsin Milwaukee, UNITED STATES

## Abstract

Salt marshes provide a bulwark against sea-level rise (SLR), an interface between aquatic and terrestrial habitats, important nursery grounds for many species, a buffer against extreme storm impacts, and vast blue carbon repositories. However, salt marshes are at risk of loss from a variety of stressors such as SLR, nutrient enrichment, sediment deficits, herbivory, and anthropogenic disturbances. Determining the dynamics of salt marsh change with remote sensing requires high temporal resolution due to the spectral variability caused by disturbance, tides, and seasonality. Time series analysis of salt marshes can broaden our understanding of these changing environments. This study analyzed aboveground green biomass (AGB) in seven mid-Atlantic Hydrological Unit Code 8 (HUC-8) watersheds. The study revealed that the Eastern Lower Delmarva watershed had the highest average loss and the largest net reduction in salt marsh AGB from 1999–2018. The study developed a method that used Google Earth Engine (GEE) enabled time series of the Landsat archive for regional analysis of salt marsh change and identified at-risk watersheds and salt marshes providing insight into the resilience and management of these ecosystems. The time series were filtered by cloud cover and the Tidal Marsh Inundation Index (TMII). The combination of GEE enabled Landsat time series, and TMII filtering demonstrated a promising method for historic assessment and continued monitoring of salt marsh dynamics.

## Introduction

Drivers of salt marsh loss are diverse from direct anthropogenic disturbances such as reclamation for agriculture [1], and indirect factors such as replacement by mangroves [2,3], eutrophication [4], herbivory [5,6], and sea-level rise (SLR) [7, 8, 9]. For example, less than half of salt marshes are predicted to keep pace with projected SLR under the Intergovernmental Panel on Climate Change’s (IPCC) representative concentration pathway 2.6, which assumes significant reductions of CO_2_ emissions [10]. The mid-Atlantic coast is one region where accretion is unlikely to keep pace due in part to high projected rates of SLR [11], glacial isostatic adjustment, and anthropogenic processes [12].

The current and future response of salt marsh to SLR is uncertain. Recent estimates of salt marsh change have shown a slowing of loss across the Atlantic coast of the USA from 2004 to 2009 with a 0.4% reduction of estuarine emergent vegetation [13]. In contrast, estimates from specific sites have demonstrated extensive losses of salt marsh including Rhode Island, Jamaica Bay, and the Chesapeake Bay [7,14,15]. Recent projections of salt marsh change suggest salt marshes will expand if they can migrate into the uplands unimpeded by coastal development [16]. Anthropogenic action or inaction contributes to the uncertainty of the projections which, necessitates monitoring of salt marsh to identify areas of loss. *In situ* methods for monitoring salt marsh have limited ability to understand regional and global salt marsh trends or verify salt marsh models. Time series analysis of satellite remote sensing has appropriate spatial and temporal resolution to monitor and understand salt marsh change.

In the mid-Atlantic, SLR is exceeding accretion rates at many locations [10]. The characteristics of these salt marshes makes them the equivalent of canaries in the coal mine; ideal systems for studying and monitoring the effect of SLR on salt marsh resilience. Many mid-Atlantic salt marshes have microtidal ranges and low sediment budgets. These characteristics increase the risk of loss to SLR [17]. The limited sediment supply of the mid-Atlantic coastal salt marshes, composed predominantly of *S*. *alterniflora* or *S*. *patens*, results in peat dominated wetlands [18], i.e., salt marshes which rely primarily on organic matter to build elevation, as opposed to those along the southeast U.S. coast, which accrete mostly mineral material [19]. Peat dominated salt marshes adapt more slowly to SLR [20]. Mid-Atlantic salt marsh characteristics such as tidal range, soil material, subsidence, and human disturbance, elevate the risk of SLR to the regions salt marsh.

A variety of remote sensing data have been applied to evaluate wetland change including very high-resolution (VHR) satellite imagery [21], Landsat [22], Synthetic Aperture Radar (SAR) [23], and aerial imagery [7]. Time series analysis of salt marshes has been conducted with many sensors including the Moderate Resolution Imaging Spectroradiometer (MODIS) [24], SPOT-5 [25], and the Landsat archive [26,27]. Google Earth Engine (GEE) has enabled time series, analysis in freshwater wetland change analysis [28]. Cloud computing and High-Power Computing are frequently employed in time series studies to quantify ecological processes, and land cover land use change (LCLUC) [29–31]. GEE facilitates our ability to understand LCLUC at regional and global scales. The utilization of these methods in salt marsh landscapes can further clarify how and where these ecosystems are changing.

Remote sensing of salt marsh is prone to time series outliers due to tidal inundation, extreme water events, and atmospheric anomalies. The tidal stage at the time of image acquisition can directly impact the extent of salt marsh vegetation in Landsat imagery [32] and VHR imagery at high tide when portions of the low marsh are submerged [21]. Time series outliers can alter the attributes and the results of an analysis [33]. Therefore, the effect of tidal outliers is a concern in remote sensing of salt marsh. The tidal marsh inundation index (TMII) has been successfully used to identify inundated pixels and improve time series results for MODIS [34]. Season and trend decomposition of the time series is another way to minimize the effect of outliers, the method is robust to noise when detecting changes greater than 0.1 Normalized Difference Vegetation Index (NDVI) [35]. In this study, filtering and seasonal and trend decomposition mitigated the effect of tidal inundation on the time series. This study innovates by applying a time series approach to aboveground green biomass (AGB) estimates derived from remote sensing to clarify long-term change of the salt marsh at a regional scope.

This study explores the capacity of time series analysis to help understand salt marsh dynamics in association with locations of stability, gradual loss, change driven by disturbance, or a combination of loss and recovery and the sources of change such as interior drowning, edge erosion, barrier island migration processes, and shifts in vegetation composition. The objectives of this study include: (1) to evaluate the salt marsh AGB estimates with high spatial resolution imagery and *in situ* biomass samples; (2) to model the change in AGB of mid-Atlantic salt marshes from 1999 to 2018 and (3) to test the TMII for use with GEE enabled Landsat time series.

## Methods

### Study site

**Fig 1:**
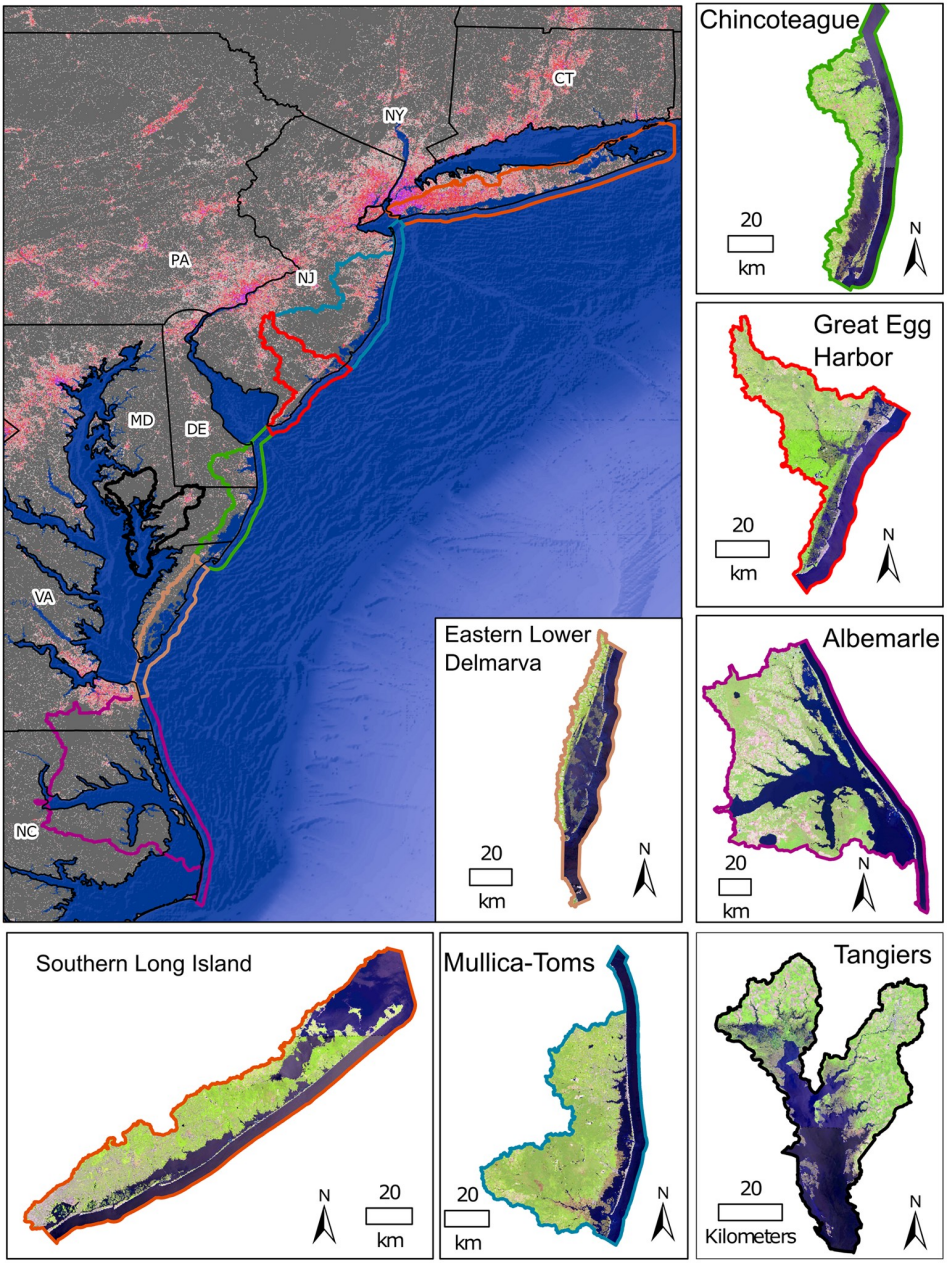
The seven HUC-8 watersheds located across the mid-Atlantic coast. Background data in display are 100 m impervious surface [40] and 30 arc-second GEBCO bathymetry data [41]. Watershed subsets are true color displays of the Landsat 8 imagery courtesy of the U.S. Geological Survey with HUC-12 watershed outlines in grey. Color outlines match watersheds in the overview to each watershed inset.

The mid-Atlantic coastal region has a variety of estuaries and bays including drowned river valleys such as the Chesapeake and Delaware Bays and barrier island lagoon systems such as Great South Bay and Barnegat Bay. Watersheds were used as the spatial extents for this study because salt marshes are affected by their watershed’s sediment supply [36] and nutrient loads [4]. The study selected USGS Hydrological Unit Code 8 (HUC-8) watersheds. This study, covered coastal watersheds across the southern sections of Long Island, NY, New Jersey, Delaware, Maryland, Virginia, and northern North Carolina (Fig 1). The majority of these watersheds are dominated by back-barrier lagoon systems with extensive salt marshes. The exception was the Tangier watershed within the Chesapeake Bay which is a drowned river valley. The Tangier watershed is an area of extensive land loss due to SLR, low sediment load, and groundwater withdrawal [22]. The dominant salt marsh species in these watersheds are *S*. *alterniflora* in the low marsh and *Juncus gerardii*, *S*. *patens*, *Distichlis spicata*, and *J*. *roemerianus* in the high marsh. Extensive changes in the mid-Atlantic are projected from climate change including shifts in salt marsh plant composition and extent, displacement of species [37], increases in decomposition rates leading to a reduction of organic accretion in the low marsh [38], and possible reductions in belowground biomass due to earlier senescence of *S*. *alterniflora* [39].

### Data

Landsat 7 and Landsat 8 Tier-1 imagery accessible with GEE were used for the time series analysis. Multispectral Landsat 7 Enhanced Thematic Mapper + (ETM+) has a 30 m spatial resolution for bands 1–5 and 7. The panchromatic band 8 has a 15 m spatial resolution. Landsat 8 Operational Land Imager (OLI) has a 30 m spatial resolution for bands 1–7 and 9. The OLI panchromatic band 8 has the same 15 m spatial resolution as the ETM+ panchromatic band.

**Fig 2:**
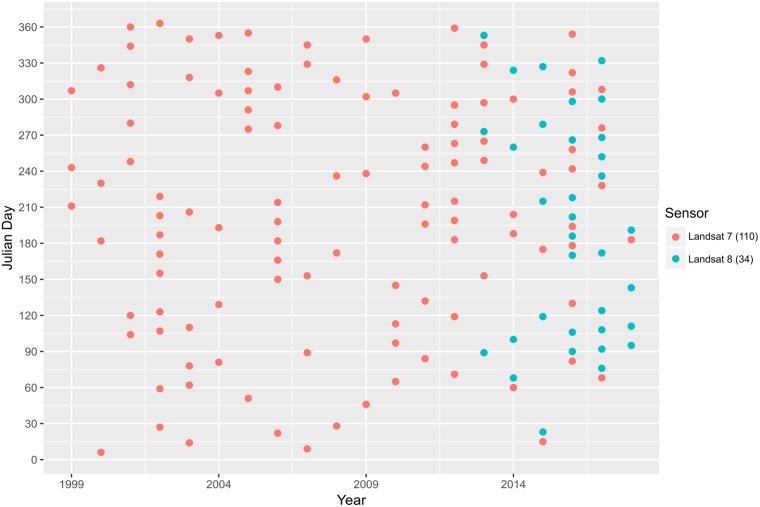
The year, Julian date, and Landsat sensor of each image after filtering by pixel cloud cover and TMII for a single Southern Long Island watershed time series.

**Fig 3:**
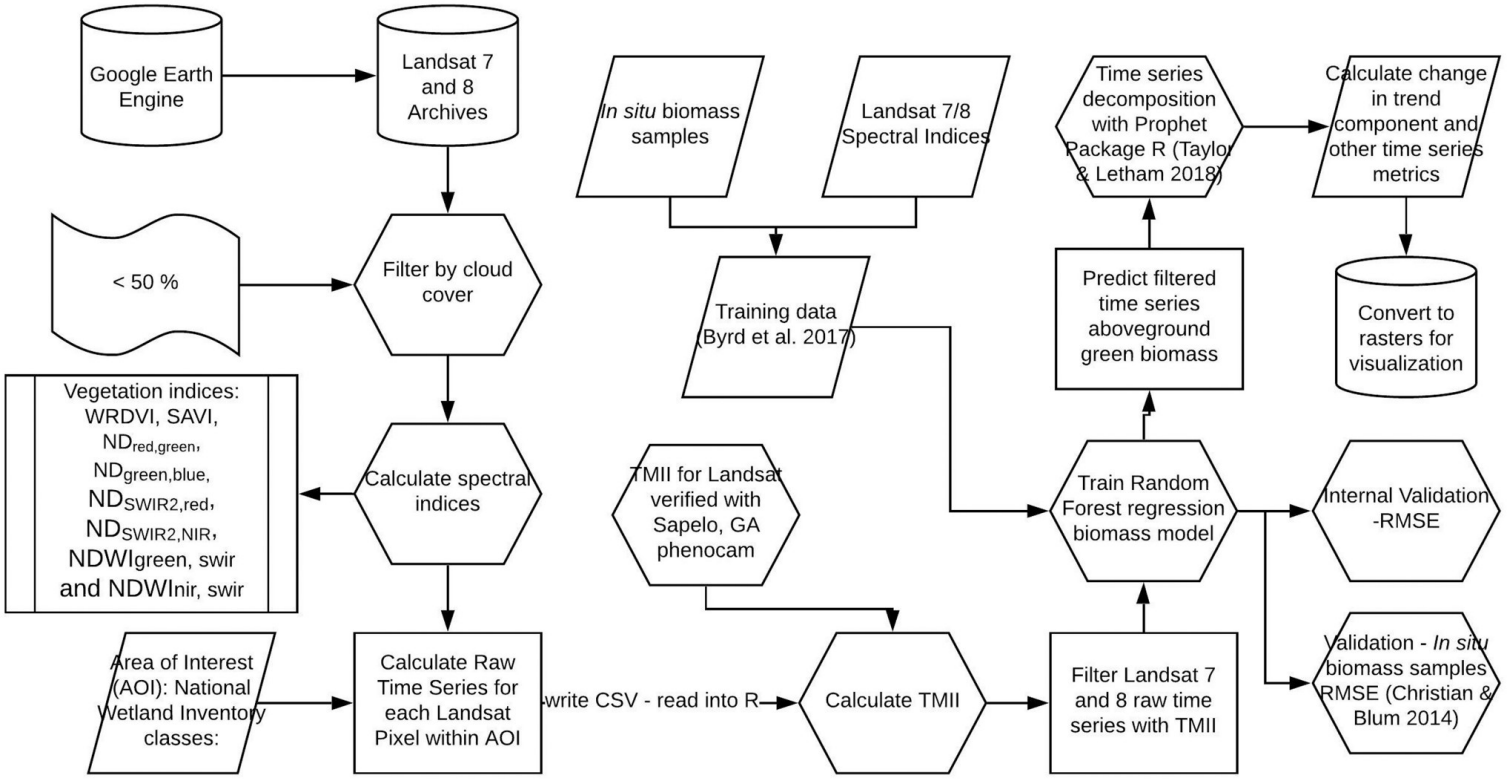
Diagram of the study’s GEE data processing, AGB model, verification, and time series analysis.

The selected ETM+ imageries were acquired from 7/01/1999 to 4/01/2017. The OLI imageries were acquired 3/20/2013–7/28/2018. The HUC-8 watersheds are covered by Landsat scenes of WRS-2 Path/Row 14/34, 14/33, 13/32, 13/31, 14/32, and 14/35. The selection and filtering resulted in a variable number of scenes per pixel across the study sites, e.g. the pixel in the Southern Long Island study area had 144 scenes (Fig 2). The average number of scenes after filtering for each watershed was 173.5, 173.0, 139.5, 169.7, 141.5, 146.3, and 170.2 for the Eastern Lower Delmarva, Tangier, Southern Long Island, Chincoteague, Mullica-Toms, Great Egg Harbor, and Albemarle, respectively. GEE was used to convert Landsat 7 surface reflectance to Landsat 8 surface reflectance following the methods in [42]. The transformed values were then used to calculate vegetation indices, Wide Dynamic Range Vegetation Index (WDRVI), Soil Adjusted Vegetation Index (SAVI), Normalized Difference Red Green, Normalized Difference Green Blue, Normalized Difference SWIR2 Red, Normalized Difference SWIR2 NIR, Normalized Difference Water Index_green, swir_ (NDWI_green, swir_), and NDWI_nir, swir_ utilized in the tidal filtering and random forest regression estimating AGB [43]. Raw time series of the spectral indices were computed for each pixel within the defined extent of salt marsh and exported from GEE (Fig 3).

National Wetland Inventory (NWI) data were used to select all pixels within the estuarine emergent vegetation class. The use of the NWI to constrain the analysis ensured that biomass models were applied to only salt marsh pixels. Pixel centroids which fell directly within creeks, ditches, and mapped pools were also excluded, resulting in the removal of some partial salt marsh pixels from the analysis. Some concern surrounds the use of the NWI layers e.g. an Illinois field assessment found they omit many wetlands [44]. All of our study areas have been updated since being mapped in the 1970/1980s, and had image acquisition dates between 2000–2015. The exception being the Albemarle watershed which had a range of image dates between 1977–2015, however, salt marshes were only in areas mapped between 1990–2015. A linear regression model was used to compare image acquisition date and average within watershed change rates.

VHR satellite imagery, e.g., Worldview-2 data, were used to verify the relationship of AGB estimates and vegetation extent. The Worldview-2 data were collected on October 11 and October 16, 2016, for the Chincoteague watershed. This imagery covered the entirety of Assateague Island. Multispectral Worldview-2 imagery possesses 2.4 m spatial resolution and a panchromatic band of 0.46 m. The spectral coverage includes 8 bands ranging from coastal blue, blue, green, yellow, red, red edge, to near infrared.

### Landsat tidal marsh inundation index

Many spectral indices such as the Enhanced Vegetation Index share formulas between Landsat and MODIS. TMII was developed for MODIS data. This study assessed the index for use with Landsat data. NDWI_green, swir_, and NDWI_nir, swir_ were calculated for each salt marsh pixel. The NDWI_nir_, _swir_ was averaged for each month across each pixel’s time series for a single sensor. The monthly mean replaced the rolling average of the MODIS TMII which included 44 temporally adjacent images [34]. Replicating such a rolling average would not be reasonable for our coarse temporal resolution. The adapted formulas and the original MODIS formulation are shown below.

1. MODIS TMII (Eq 1)
  TMII=(1-(1/e^(0.3+16.6*NDWI4,6-25.2*rollingmean(NDWI2,5))) (1)
  [34].
2. Landsat 7 TMII (Eq 2)
  TMII=(1-(1/e^(0.3+16.6*NDWI2,5-25.2*monthlymean(NDWI4,5))) (2)
3. Landsat 8 TMII (Eq 3)
  TMII=(1-(1/e^(0.3+16.6*NDWI3,6-25.2*monthlymean(NDWI5,6))) (3)

The resulting index was evaluated at the Sapelo Island, GA phenocam across Landsat 7 and Landsat 8 images from WRS-2 Path/Row 16/38 and 17/38 and a date range from 8/09/2013 to 5/03/2018. The evaluation followed the approach of [34] i.e. verifying if the salt marsh visible from the phenocam was inundated or not during a Landsat image acquisition.

### Above ground biomass model

Vegetation indices are frequently used in time series analysis, including monitoring forest disturbance [45], rice distribution [46], agricultural abandonment [47], and salt marsh change [48]. NDVI and many related vegetation indices (i.e. WRDVI and SAVI) are indicators of aboveground biomass [49]. Recent methods for estimating AGB in freshwater and salt marsh environments have relied on vegetation indices [43,50]. This method allows for the estimation of AGB for the majority of plants common in the estuarine emergent wetland category of [51]. By utilizing AGB instead of vegetation indices this study seeks to communicate to a wider interdisciplinary audience.

The spectral indices were converted to AGB following the methods put forth in [43] which achieved a RMSE of 310 g/m^2^ and R^2^ = 0.59, for calculating AGB with Landsat data. A training data set of *in situ* biomass and corresponding Landsat spectral indices (n = 2400) were collected from 2005 to 2015 in San Francisco Bay, the Everglades, Louisiana, Cape Cod, Puget Sound, and Chesapeake [52]. A random forest model was trained utilizing a subset of the training data to control for over representation of the San Francisco Bay and Everglades training points [43]. This study trained a model following the same approach and achieving similar results, however, given the random downsampling model performance varied slightly.

Further verification of the model was performed using end of season *in situ* biomass estimates from 1999–2014 for the Eastern Lower Delmarva and Chincoteague Watersheds [53]. These samples were outside the area previously sampled and represent the transferability of the model to the additional watersheds. The aboveground biomass estimates included 16 sites at Mill Creek, Bellvue, Steelman’s landing, Gator Track, Cushman’s landing, Oyster Marsh, Indian Town, Box Tree, Brownsville, Hog Island north, Hog Island south, Kegotank, Green Creek, Wallops Island, Woodland Farm, and Assateague [53]. The sites were sampled along transects at four locations, creekside, low marsh, high marsh, and upland transition [53]. Replicates from each location were averaged to get an estimate of each sites aboveground biomass in a single year which was then compared to the average AGB estimates for July, August, and September in the corresponding years. RMSE was calculated considering each year at each site, and comparing the average across all years for each site.

### Time series analysis and statistical analysis

The time series analysis was conducted on Landsat 7 and 8 scenes filtered by cloud cover <50%, pixel quality, and a TMII value of >0.2. Landsat 5 data were not utilized in this study due to the lack of both a conversion method into Landsat 8 surface reflectance and verification of the AGB model [42, 43].

The R package Prophet was used for time series analysis [54]. The seasonal-trend decomposition method uses locally weighted regression smoother (LOESS) to isolate the seasonality, trend, and noise [55]. The approach has been used for many remote sensing time series studies [30,35,56]. The prophet package was used due to its robustness to irregular time series, ability to calculate many time series and identify trends and seasonality. All measures of change were derived from the time series analysis using the trend component i.e. trend end–trend start resulting in time series derived measure of change in AGB.

The effect of tidal range on salt marsh change was explored with the use of data from NOAA tidal stations. The tidal ranges of each tidal station within the study area were interpolated into a raster map of tidal ranges as they coincided with HUC-12 watersheds within the study area (Fig 1). All Landsat centroids that were in the interior of the salt marsh, i.e., >30 m from an edge, were analyzed. The effect of tidal range on average change in AGB across HUC-12 watersheds for the four most prevalent salt marsh classes, i.e., *estuarine emergent regularly flooded*, *estuarine emergent irregularly flooded*, *estuarine emergent ditched regularly flooded*, and *estuarine emergent ditched irregularly flooded*, were compared with linear regression. The average change in AGB was compared to the average tidal range. The Albemarle watershed, NC was excluded from the analysis due to the large gaps between tidal stations. Each HUC-12 watershed also had the change rate for edge pixels and interior pixels compared. Edge pixels were those within 20 m of the NWI polygon boundary. Interior pixels were those further than 20 m from the NWI polygon boundary.

An analysis of all Landsat pixels of the *estuarine emergent regularly flooded*, *estuarine emergent irregularly flooded*, *estuarine emergent ditched regularly flooded*, and *estuarine emergent ditched irregularly flooded* classes were conducted for each HUC-8 watershed. Kruskal-Wallis and post-hoc Dunn’s test with Bonferroni adjustment compared the trend in AGB from 1999 to 2018 for each watershed across these four salt marsh classes. Kruskal-Wallis is a non-parametric comparison on ranks [57], pairwise comparisons between classes were conducted using Dunn’s test [58].

Worldview-2 image classification of interior salt marsh mudflats was used to assess the relationship of AGB estimates and vegetation extent within the test pixel. The Wordlview-2 image classification was an object-based image analysis [14,21]. This analysis was conducted for a portion of the salt marsh on the Maryland side of Assateague Island within the Chincoteague watershed. This analysis was conducted for mudflats on Assateague Island which corresponded with WRS-2 Path/Row 14/33.

## Results

### Tidal marsh inundation index

**Fig 4:**
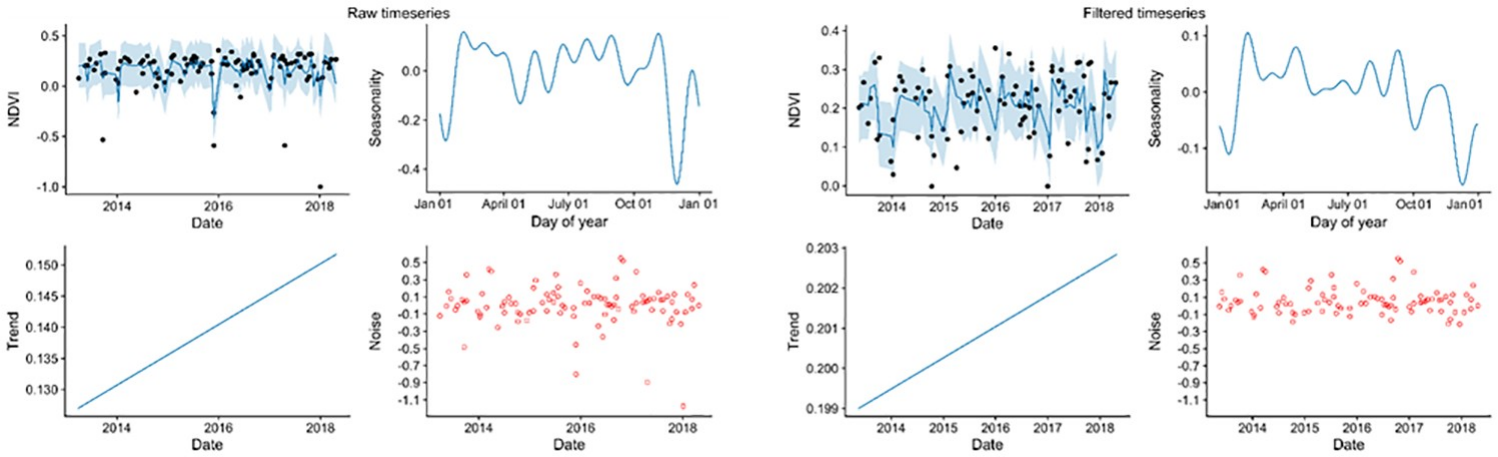
Evaluation of TMII with time series analysis using Landsat 7 and 8. Raw time series includes inundated dates. Filtered time series was excluded dates with TMII > 0.2.

The TMII was assessed by evaluating the inundation of each Landsat image date and time of collection at the phenocam and by plotting the decomposed time series before and after filtering (Fig 4). The filtered time series removed all pixels with a TMII >0.2. This level of TMII was suggested previously and performed well in the analysis with the phenocam. The filtered time series removed extreme outliers reduced the observed trend and improved the seasonal graph. The phenocam analysis had a limited number of inundated scenes to work with using images from both WRS-2 Path/Row 16/38 and 17/38. For Landsat 7 and 8, the phenocam image evaluation verified that 10 of the 14 images with TMII >0.2 were inundated. The performance improved slightly when just considering the Landsat 8 imagery, which found 7 out of 9 inundated images were correctly identified. The index had few false negatives for inundation with 148 out of 150 non-inundated images being accurately determined. The filter was applied due to its ability to remove outliers and improve both the seasonal and trend component of the time series decomposition (Fig 4).

### Biomass model and change

**Fig 5:**
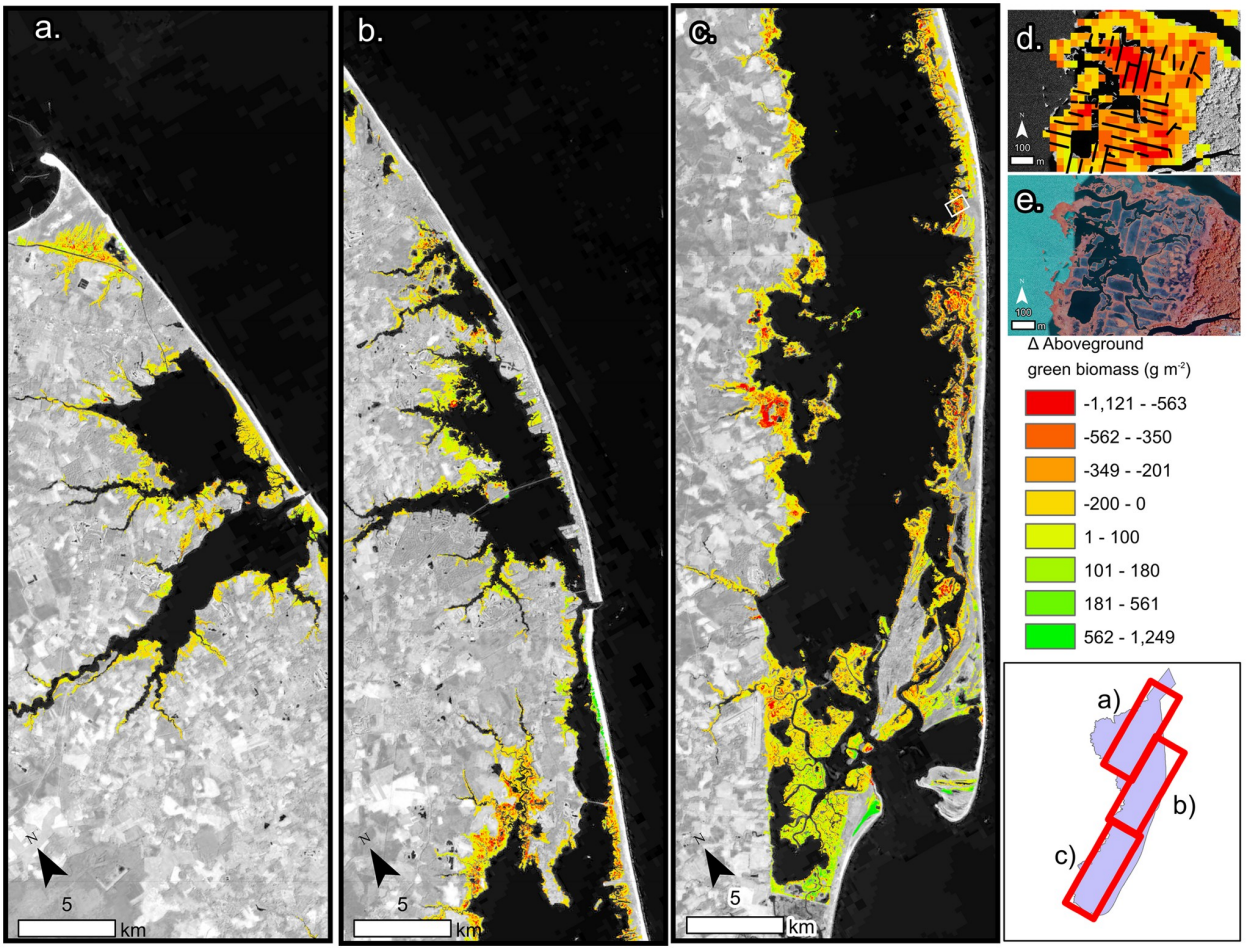
a-c. Change in AGB from 1999–2018 for the Chincoteague watershed, encompassing the eastern shore of Maryland and a section of Virginia and Delaware. Background image Landsat 8 courtesy of the U.S. Geological Survey d. Inset (white box in c.) of salt marsh change and mosquito ditches. e. 2018 NAIP imagery courtesy of the U.S. Geological Survey in pseudo-color image of the same extent as d.

**Fig 6:**
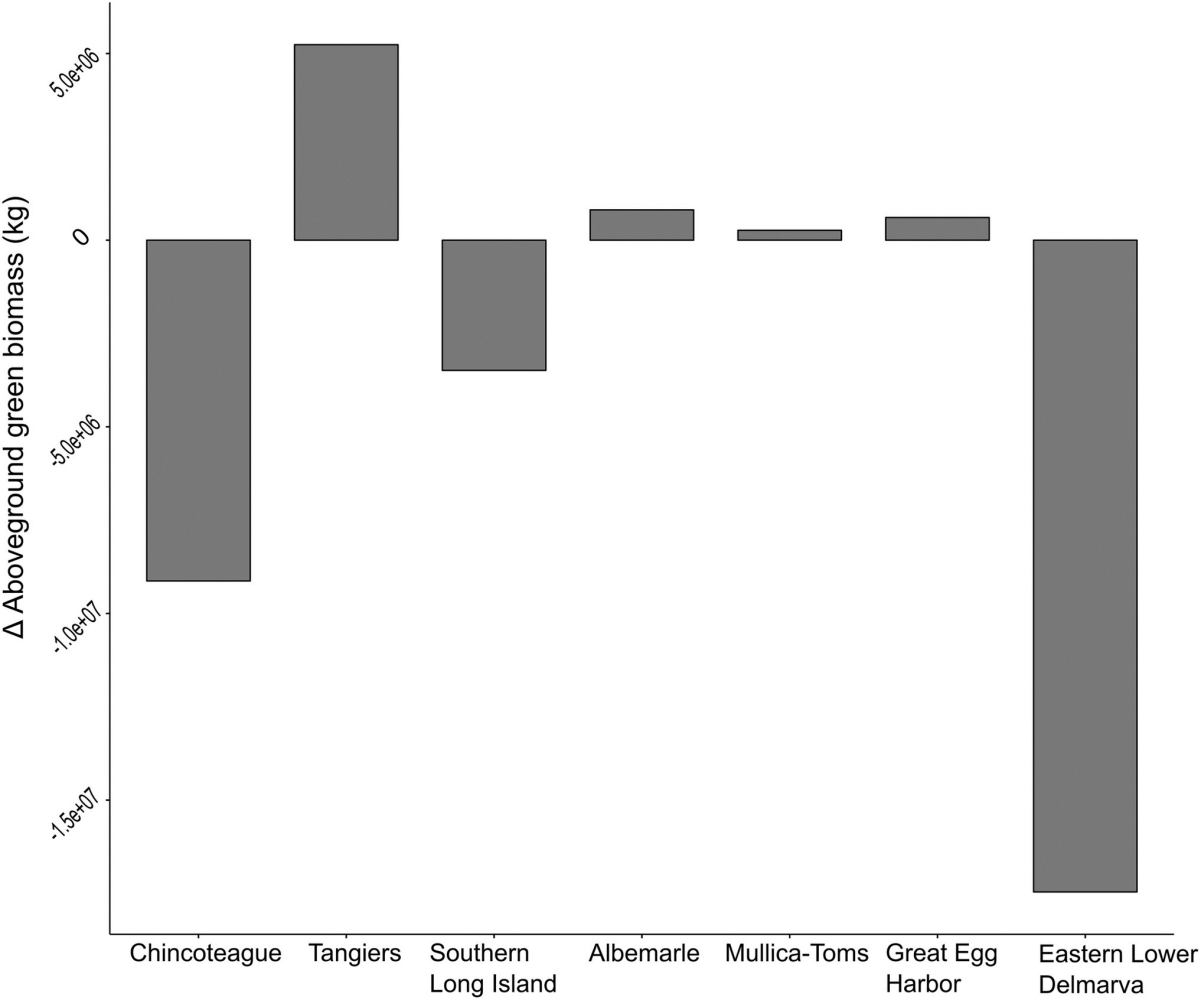
The net change (1999–2018) in AGB for each watershed.

The ability of the time series trend component to reveal salt marsh change was evident in the identification of both losses and gains across the watersheds. Across the studied watersheds 52% of salt marsh experienced a decline in AGB with an average change of -17 g/m^2^ (Table 1). In the Chincoteague watershed, declines were common, and interior loss along the back-barrier of Assateague Island National Seashore was apparent (Fig 5). Increases in AGB were most prominent in the prograding areas to the south of Assateague Island (Fig 5c) and on the overwash fans on northern Assateague Island (Fig 5b). Chincoteague, Eastern Lower Delmarva, and Southern Long Island all had moderate declines in biomass (Table 1). Tangiers, Mullica-Toms, Albemarle, and Great Egg Harbor had slight increases. The Chincoteague, Eastern Lower Delmarva, and Southern Long Island watersheds demonstrated considerable net loss of AGB (Fig 6). The Eastern Lower Delmarva watershed had the largest average loss which was -67 g/m^2^. The Tangier watershed had the largest average gain which was 15 g/m^2^.

**Table 1:** The percentage of change, total area, and mean trend of estuarine emergent irregularly flooded, estuarine emergent regularly flooded, estuarine emergent irregularly flooded ditched, and estuarine emergent regularly flooded ditched classes from 1999 to 2018.

HUC 8 Code	Name	Decrease (%)	Increase (%)	Area (hectares)	Mean trend (g/m^2^)
02080110	Tangier	35	65	35650	15
02030202	Southern Long Island	76	24	7226	-48
02040301	Mullica-Toms	48	52	18891	1
02040302	Great Egg Harbor	49	51	21172	3
02040303	Chincoteague	62	38	14538	-63
02040304	Eastern Lower Delmarva	75	25	25880	-67
03010205	Albemarle	40	60	16223	5
	Mid-Atlantic coast	52	48	139580	-17

The *in situ* analysis resulted in a site-wide RMSE of 144±7 g/m^2^ with the confidence interval resulting in a conversion factor from wet biomass to dry of between 0.55 and 0.6. The *in situ* yearly RMSE for the Eastern Lower Delmarva watershed 1999 to 2014 was found to be 298 g/m^2^ ±15. This RMSE compares favorably with the RMSE calculated internally 310 g/m^2^ [43], and arrived at by this study (RMSE of 350 g/m^2^ ±16, R^2^ = 0.62). The areas of uncertainty include the exact location of the sampling sites and differences between dates of the end of season sampling and July, August, and September satellite estimates.

### Time series trend and statistical analysis

**Fig 7:**
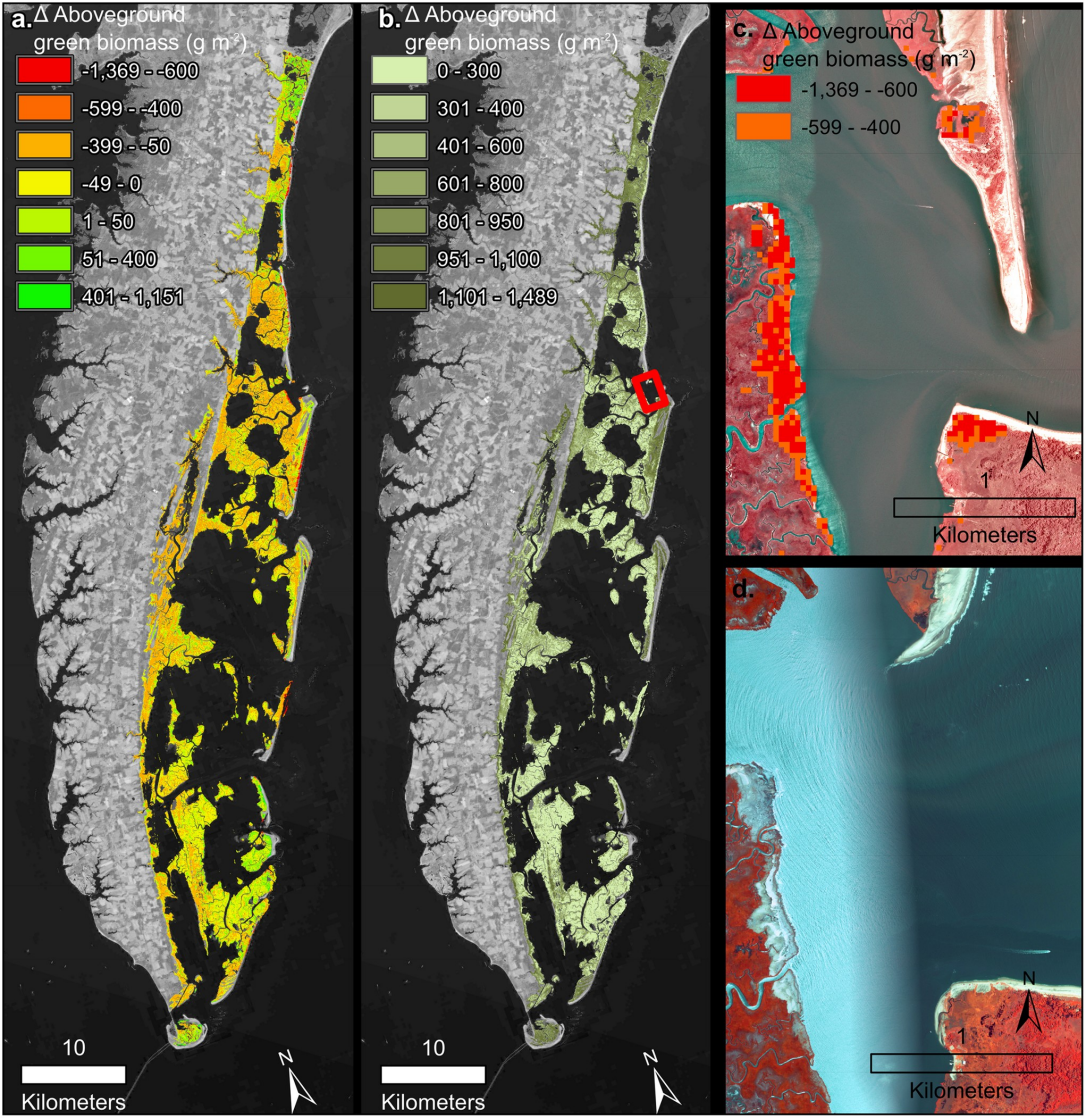
a) Eastern Lower Delmarva watershed change in AGB from 1999 to 2018. b) Eastern Lower Delmarva watershed with the average AGB in July, August, September of 2017. Background imagery Landsat 8 courtesy of U.S. Geological Survey c) Salt marsh trend for an area of loss (2014–2016), NAIP image from 2012 courtesy of the U.S. Geological Survey. d) NAIP 2016 image following barrier spit change.

**Fig 8:**
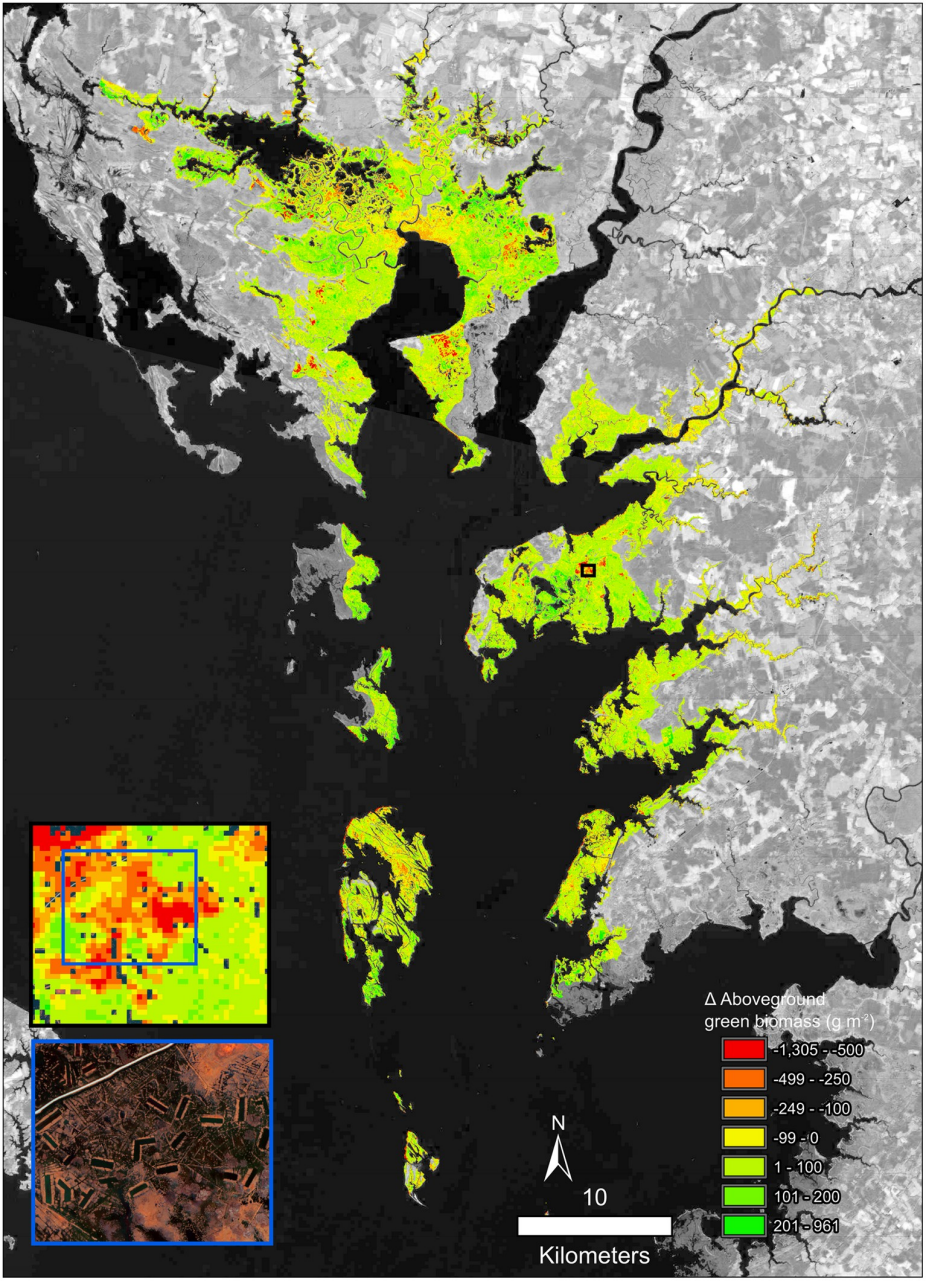
Change in AGB from 1999 to 2018 in the Tangier watershed. a. Shows an inset area of concentrated change in the AGB trend. Background imagery Landsat 8 courtesy of U.S. Geological Survey. b. shows a subset of the heavily ditched area with pseudo color NAIP imagery from 6/1/2017.

The NWI acquisition dates effect on change rate for the area was determined across all watersheds. No relationship between acquisition date and change rate was found F(_1,18_) = 0.67, *p* = 0.42) and R^2^ = -0.02. Instead the rates of change varied greatly both across watersheds and within a watershed. The analysis with Moran’s I for each of the watershed confirmed clustering of salt marsh change within all watersheds (Table 2). In particular, the Eastern Lower Delmarva watershed, which had the largest total loss, has very evident clusters of loss (Figs 6 and 7). Trend maps reveal a clustering of loss around landscape features such as ditches, inlets, and rivers even in stable watersheds (Fig 8).

**Table 2:** The results of the Moran’s I test of spatial autocorrelation for each of the watersheds. The neighbor distance was 200 m across all watersheds.

Watershed	Moran’s Index	*P* value	z-score
Tangier	0.39	< 0.001	1572
Southern Long Island	0.41	< 0.001	1319
Mullica-Toms	0.53	< 0.001	1509
Great Egg Harbor	0.34	< 0.001	1050
Chincoteague	0.57	<0.001	1252
Eastern Lower Delmarva	0.45	<0.001	1513
Albemarle	0.41	<0.001	1319

**Fig 9:**
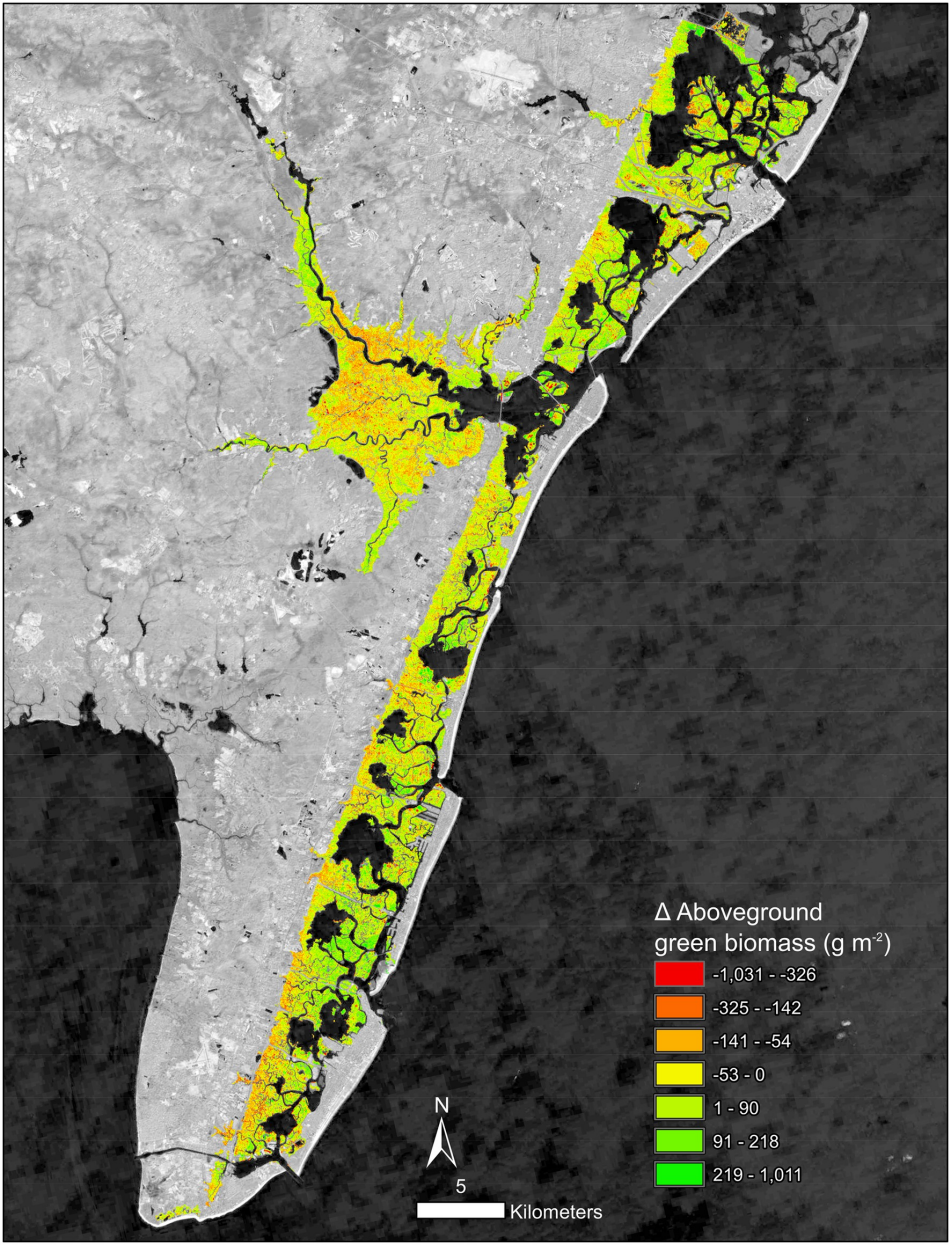
Great Egg Harbor watershed, stretching from Cape May, NJ to just south of Great Bay, NJ. The change of AGB from 1999 to 2018. Background imagery Landsat 8 courtesy of U.S. Geological Survey.

**Fig 10:**
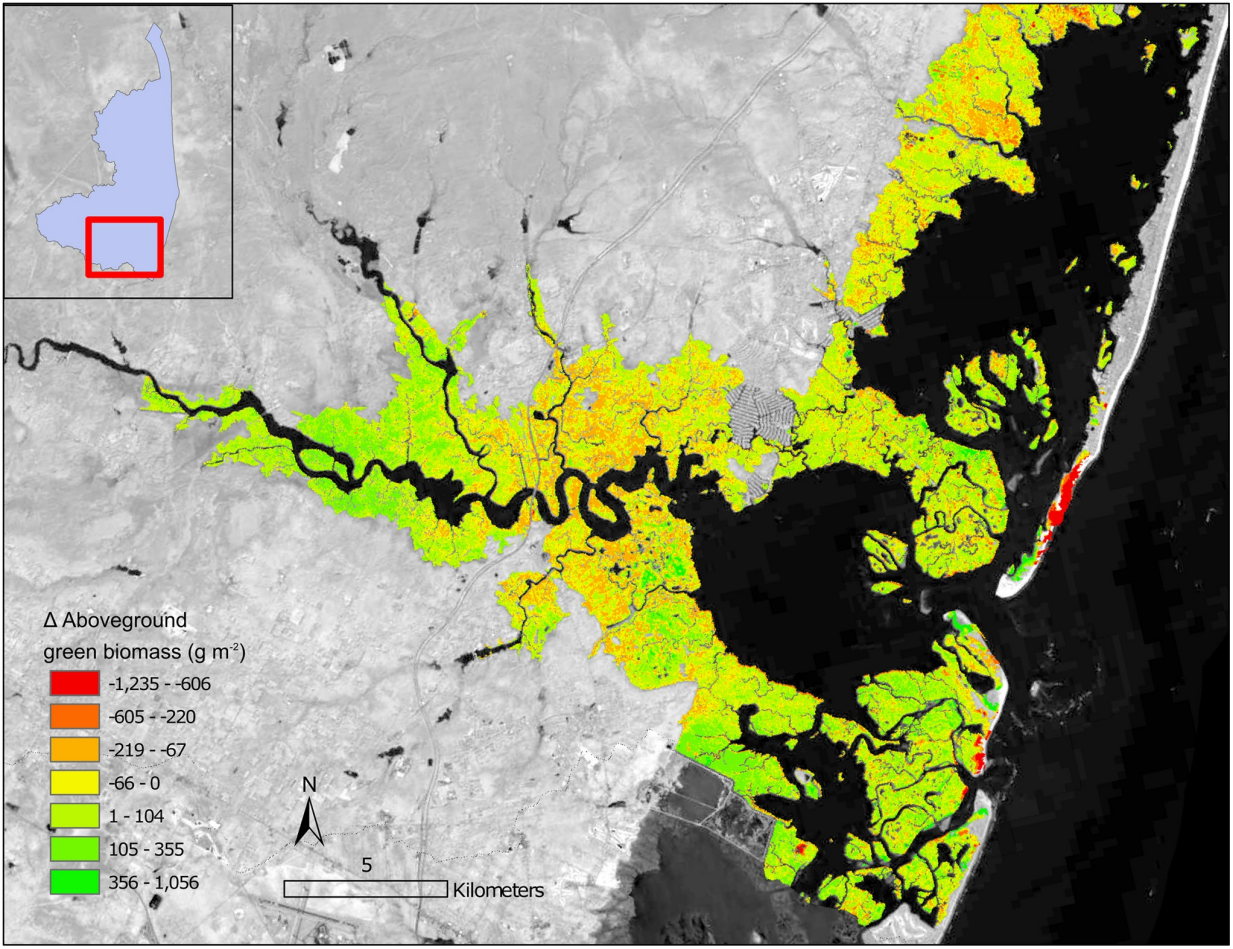
Change in AGB from 1999–2018 for an area surrounding Great Bay, NJ, a section of the Mullica-Toms watershed. Background imagery Landsat 8 courtesy of U.S. Geological Survey.

Kruskal-Wallis test was used to test the difference between dominant salt marsh types with each analysis finding significant differences (Table 3). Dunn’s *post hoc* test determined that Chincoteague and Albemarle were the only watersheds were ditched regularly flooded marshes lost vegetation at a lesser rate than regularly flooded salt marshes. Eastern Lower Delmarva and Tangiers were the watersheds where the regularly flooded salt marsh lost more biomass than the irregularly flooded salt marsh. Mullica-Toms, Great Egg Harbor, and Tangier watersheds were the watersheds to demonstrate a small increase in AGB. These watersheds were mosaics composed of a combination of increases and decreases in AGB (Figs 8, 9 and 10).

**Table 3:** The results of the Kruskal-Wallis and Dunn’s post hoc test for each of the 7 watersheds. The tests compared the four most common estuarine emergent vegetation subclasses including irregularly flooded (E2EM1N), regularly flooded (E2EM1P), ditched irregularly flooded (E2EM1Nd), ditched regularly flooded (E2EM1Pd).

Watershed	Kruskal-Wallis	Dunn’s post hoc test					
		regularly flooded vs. ditched regularly flooded	regularly flooded vs. irregularly flooded	ditched regularly flooded vs. irregularly flooded	regularly flooded vs. ditched irregularly flooded	ditched regularly flooded vs. ditched irregularly flooded	irregularly flooded vs. ditched irregularly flooded
Tangier	H(3) = 1239, *p* < 0.001	*Z* = 11.9 *p* < 0.001	*Z* = -27.3 *p* < 0.001	*Z* = -15.4 *p* < 0.001	*Z* = -16.5 *p* < 0.001	*Z* = -16.5 *p* < 0.001	*Z* = -13.9 *p* < 0.001
Southern Long Island	H(3) = 248, *p* < 0.001	*Z* = 9.0 *p* < 0.001	*Z* = 8.5 *p* < 0.001	*Z* = -3.9 *p* = 0.001	*Z* = 14.4 *p* < 0.001	*Z* = -0.4 *p* = 1.00	*Z* = 8.2 *p* < 0.001
Mullica-Toms	H(3) = 3099, *p* < 0.001	*Z* = 14.5 *p* < 0.001	*Z* = 2.5 *p* = 0.04	*Z* = -14.0 *p* < 0.001	*Z* = 36.9 *p* < 0.001	*Z* = 5.7 *p* < 0.001	*Z* = 47.2 *p* < 0.001
Great Egg Harbor	H(3) = 4166, *p* < 0.001	*Z* = 13.8 *p* < 0.001	*Z* = 4.1 *p* <0.001	*Z* = -12.8 *p* < 0.001	*Z* = 36.1 *p* < 0.001	*Z* = 6.1 *p* < 0.001	*Z* = 57.9 *p* < 0.001
Chincoteague	H(3) = 1280, *p* < 0.001	*Z* = -5.3 *p* < 0.001	*Z* = 2.1 *p* = 0.1	*Z* = 6.8 *p* < 0.001	*Z* = 28.2 *p* < 0.001	*Z* = 28.2 *p* < 0.001	*Z* = 23.4 *p* < 0.001
Eastern Lower Delmarva	H(2) = 2262, *p* < 0.001	NA	*Z* = -47.5 *p* < 0.001	NA	*Z* = 2.3 *p* = 0.04	NA	*Z* = 4.5 *p* < 0.001
Albemarle	H(3) = 2142, *p* < 0.001	*Z* = -31.6 *p* < 0.001	*Z* = 14.7 *p* < 0.001	*Z* = 39.3 *p* < 0.001	*Z* = 19.9 *p* < 0.001	*Z* = 43.9 *p* < 0.001	*Z* = 1.6 *p* = 0.36

No significant effect of the tidal range was found for the entirety of the average AGB change by HUC-12 watersheds (F(_1,573_) = 0.52, *p* = 0.52) and R^2^ = 0. However, when comparing those sites with irregular tidal inundation, mosquito ditches, and a tidal range < 0.8 m; then sites with small tidal ranges saw significantly more loss (F(_1,34_) = 6.2, *p* < 0.05) and R^2^ = 0.16). When comparing those sites with regular tidal inundation, mosquito ditches, and a tidal range < 0.8 m; then small tidal ranges also saw significantly more loss (F(_1,14_) = 7.1, *p* < 0.05) and R^2^ = 0.33). Neither inundation regime without mosquito ditches had a significant relationship to tidal range.

The relationship of Landsat derived estimates of AGB and salt marsh extent were verified with Worldview-2 image classification of salt marsh on Assateague Island National Seashore [59]. The Worldview-2 classification was used to compare non-vegetated extent within a pixel to the estimates of AGB. This comparison found a negative relationship between biomass estimates and the area of mudflat within a pixel (F(_1165,1_) = 1316, p < 0.001) and R^2^ = 0.53. The verification with VHR imagery suggests that the Landsat AGB is related to vegetation extent.

## Discussion

The *in situ* AGB samples from the Eastern Lower Delmarva verify a similar accuracy to internal out-of-box accuracy assessments from the Random Forest model. A RMSE of 310 g/m^2^ was achieved in [43] compared to this study’s out of box estimate of 350 ±16 g/m^2^. However, models have been observed to perform better at the site scale [50]. The site-wide RMSE, compared site averages for all available years, was 144±7. The yearly comparison between *in situ* samples and Landsat estimates had high variability in part due to different resolutions i.e., *in situ* samples were a much finer resolution (0.0625 m^2^) than a Landsat pixel (900 m^2^). More *in situ* samples in the site-wide aggregate resulted in an improved RMSE.

**Fig 11:**
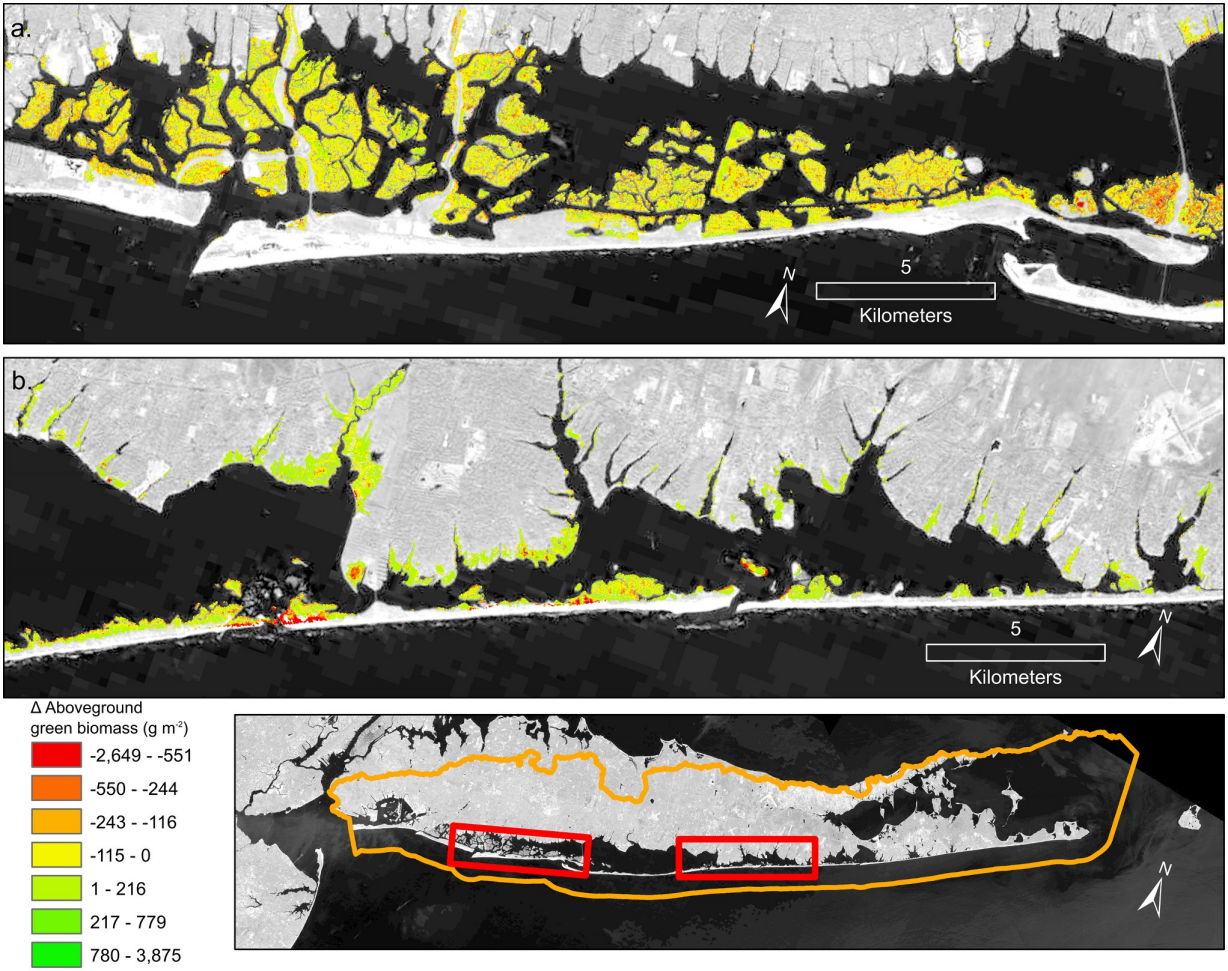
Two subsets of the Southern Long Island watershed. Change in AGB from 1999–2018: a) the back bay salt marshes of Jones Beach Island; b) the north-eastern section of Fire Island and Moriches Bay. Background imagery Landsat 8 courtesy of U.S. Geological Survey.

AGB loss includes several processes observed in the high-resolution imagery and time series: 1) interior loss and fragmentation, 2) salt marsh loss due to inlet widening and change, 3) edge erosion, and 4) overwash (Fig 11). Additional processes such as the conversion of high marsh to low marsh likely occurred but require additional in situ data to identify. Migration into the upland was outside the scope of this study. However, future studies should pursue monitoring both these components of salt marsh change.

AGB declined in three of the study watersheds. Clusters of significant loss were even evident in stable watersheds (Figs 8, 9 and 10). One possible explanation for the variability in watershed-wide change is tidal range. The analysis of tidal range makes it clear that ditched salt marshes with < 0.8 m tidal range were more prone to loss of AGB than the relatively more stable areas (> 0.8 m). This result is supported by previous models which found for the same suspend sediment concentrations macrotidal marshes (>4m tidal range) can adapt to much higher rates of SLR than microtidal (<2 m tidal range) salt marsh [60]. The tidal range and mosquito ditches were some of the site-specific factors which drove loss at the sub-watershed scale. These patterns agree with the literature which suggests long-term declines at the local scale [7,14,15] and projected salt marsh stability until the late 21st century under conservative estimates of SLR [60].

The higher rates of loss in ditched tidal range marshes could be related to the filling of mosquito ditches which has been identified as a possible contributing factor to salt marsh dieback and loss of *Spartina patens* in Rhode Island [61]. The fragility of these microtidal marshes is likely due to the relationship between tidal range and the growth range of *Spartina alterniflora* [62,63]. Ditched salt marshes comprised approximately 1/3 of all salt marsh pixels analyzed. The filling or removal of ditches can result in increased inundation of the salt marsh platform [64]. These salt marshes are undergoing hydrological changes that are altering vegetation extent and quantity of plant biomass.

Edge erosion was compared to interior loss finding all watersheds besides Chincoteague had a higher average rate of edge loss. In Chincoteague watershed edge areas lost on average 56 g/m^2^ compared to interior areas which lost on average 63 g/m^2^. Chincoteague’s site conditions, i.e., microtidal range and mosquito ditches, partially explained the higher rates of loss (Fig 5c; Table 3). Additionally, Chincoteague saw similar rates of loss occurring in regularly flooded and irregularly flooded salt marsh suggesting minimal differences between tidal regimes in microtidal areas (Table 3). However, in both Tangiers and Easter Lower Delmarva regularly flooded salt marsh had a greater loss than an irregularly flooded salt marsh. Despite spatial proximity, these watersheds are experiencing different change regimes.

RMSE was high on individual dates of imagery, decomposing the time series addresses much of this uncertainty by removing both the seasonal component and error isolating the trend, e.g., differencing 2018 AGB by 1999 AGB would compound the error. BFAST, a decomposition-based change detection method was robust to added noise when detecting changes [35]. Additionally, most areas had little change (< 100 g/m^2^) demonstrating the approaches ability to discern stable areas. Small declines in AGB could be the result of within pixel changes, including vegetation type, plant composition, and percent cover or some combination of these factors. For example, increased inundation can cause replacement of high marsh plants with *S*. *alterniflora*, and this is likely to reduce aboveground biomass [65]. Declines in AGB of irregularly flooded areas were possibly related to the replacement of high marsh with *S*. *alterniflora* which has been observed on Long Island [66] and Rhode Island [61]. In the mid-Atlantic, estimates of aboveground biomass for *S*. *patens*, *J*. *roemerianus*, and *S*. *alterniflora* were 1399 g/m^2^, 853 g/m^2^, and 257 g/m^2^, respectively [18]. The shift from *S*. *patens* or *J*. *roemerianus* to *S*. *alterniflora* could result in a loss of above, and presumably, belowground biomass.

The high-resolution satellite imagery analysis found that salt marsh/mudflat extent within a pixel partially explained Landsat estimates of AGB. However, vegetation extent did not explain all variation in the AGB. Other likely contributing factors include the amount of water, vegetation composition, and geometric rectification of the two datasets, in some salt marshes *Spartina alterniflora* along the marsh edge has greater aboveground biomass [67]. These differences and other site characteristics result in variability of the biomass estimates. Additional in situ verification would be necessary to establish the relationship of these changes to shifts in the vegetation community.

### Tidal filtering

The use of all available data is vital for understanding seasonal and long-term vegetation trends [29]. The time series limited temporal phases due to clouds, tides, 16-day revisit, and Landsat 7’s shutter synchronization anomalies makes keeping all quality data essential. The TMII filter is unique to the vegetation cover of a particular pixel. Therefore, it did not over filter those areas with frequent inundation. Adapting the index to Landsat posed several challenges, including different bandwidths and lower temporal resolution. The conversion of rolling to monthly averages and substitution of bands with appropriate equivalents addressed these issues (Eqs 1–3). The filtering improved time series trend estimates (Fig 4). The rarity of false positives limited any reduction of quality data while removing many inundated images. In this study, the amount of data was essential to ensure enough images were available following filtering by tides, cloud cover, and data quality. Tidal filtering is necessary to improve time series modeling of salt marsh and our understanding of long-term salt marsh change.

### Salt marsh change

There is debate about how salt marshes will change in the Anthropocene; site-level research offers conflicting insight into the relationship of SLR to salt marsh loss such as multiple stressors leading to rapid loss [68] or only extreme SLR scenarios (30 cm) resulting in drowning [69]. Modeling studies suggest migration could lead to relative salt marsh stability [16]. Due to differences in scale, these two estimates are not necessarily mutually exclusive, therefore to bridge these studies, we require regional and global studies of salt marsh change such as this one. This study found localized areas of significant salt marsh loss across all watersheds, but also relative stability when examined at the watershed scale. The mid-Atlantic salt marshes are projected to change rapidly, and this research suggests a limited change in many of the watersheds studied. The biomass model utilized in this study should be expanded and verified for additional countries were similar species exist, such as China, where both *Phragmites australis* and *Spartina alterniflora* are prevalent species with a complex relationship [70, 71]. Salt marshes are changing globally [72], with losses resulting in the release of blue carbon stores [73]. A critical carbon sink and potential source are global reasons for interest in salt marsh change. Global studies are not always possible at high spatial resolution and fine time scale. Regional studies in high-risk areas can inform our comprehensive understanding of salt marsh change.

Persistence versus die-off of salt marshes has been attributed to a variety of drivers such as sediment supply [74], edaphic characteristics of the salt marsh [75], elevation [7], nutrient enrichment [4], and basin characteristics [76]. Honeycombing of the interior salt marsh was evident particularly, in ditched salt marshes and across the Chincoteague watersheds (Fig 5d and 5e.). This relationship was most likely due to the combination of altered hydrology from mosquito ditches and small tidal ranges, causing greater vulnerability to SLR. The clustering of change in the salt marsh environments was evident visually and from the results of the Moran’s I analysis (Table 1).

The Eastern Lower Delmarva watershed, had a significant average rate of loss (Fig 6) and low average biomass, 529 g/m^2^ over July, August, and September of 2017 (Fig 7b). Barrier island migration at rates of 1–6 m yr^-1^ drives salt marsh losses in the region [77]. Sediment supply and salt marsh basin width have been suggested as drivers of salt marsh change in the Eastern Lower Delmarva [76]. The Eastern Lower Delmarva represents a different change regime than this study’s other barrier island watersheds. Migration of the seaward salt marsh boundary, minor shifts in the interior back-barrier salt marsh, and significant edge erosion due to inlet shifting was evident across the watershed (Fig 7). The time series approach was able to isolate discrete events; however, this process was not automated (Fig 7c). Temporally discrete events such as overwash or barrier spit shifts resulted in a significant reduction in percent AGB for many of the back barrier salt marshes. In the Eastern Lower Delmarva, 1% of all areas analyzed experienced a < -400 g/m^2^ loss from (Summer 2014-Summer 2016). This study demonstrates that the site’s salt marshes are low biomass, suggesting even small losses are a large percent of the sites AGB. This method has the ability to monitor salt marsh under a variety of change regimes.

## Conclusion

A combination of medium resolution imagery, time series analysis, biomass modeling, and tidal filtering were utilized to understand salt marsh change. This paper reports a new approach to tidal filtering Landsat time series data for salt marsh environments. The approach improved time series analysis in the tidally inundated areas. The combination of time series analysis and biomass models gave an improved understanding of salt marsh change. The regional study included barrier island (n = 6) watersheds across the mid-Atlantic and a sub-watershed of the Chesapeake Bay. AGB declines were identified across the study area, with a mean of -17 g/m^2^ (Table 1). In the mid-Atlantic coastal watersheds, 52% of all area analyzed declined from 1999 to 2018. Four watersheds demonstrated positive trends (> 0 g/m^2^), however minor, widespread declines due to SLR were not evident. However, clusters of loss in all watersheds were apparent. These clusters of loss corresponded with barrier island processes and interior drowning.

The methods of this study demonstrate the importance of tidally filtering the time series. Additionally, *in situ* verification of biomass estimates, and use of time series decomposition to isolate long term trends. This study conducted a completely new accuracy assessment from *in situ* data outside the training areas of the biomass models, demonstrating the model’s applicability at additional sites in the USA. The conversion to AGB is an important approach for engaging an interdisciplinary audience that may not be familiar with vegetation indices. Further, biomass is a clear indicator of salt marsh resilience, tied to ecogeomorphic feedbacks that contribute to salt marsh resilience. The current analysis demonstrates the use of AGB estimates as an indicator of salt marsh change applied to multiple watershed scales. Limitations of the method lead to the exclusion of important change processes such as migration, future work will include the development of methods to integrate migration into this methodology.

GEE created a single processing environment facilitating the filtering of Landsat images, analysis. The limiting factor for the process was exporting data from GEE to be further analyzed. The Landsat archive is the only option for decadal time series of salt marsh environments with medium spatial resolution and an extensive archive. GEE was an efficient data processing environment for the calculation of vegetation indices, the conversion of Landsat 7 surface reflectance into Landsat 8 surface reflectance, and processing of the raw time series. These methods utilized globally available remote sensing data in the form of the Landsat archive and GEE limiting the computing costs. These methods reduced hardware limitations and expand the potential geographic scope of salt marsh change analysis for both historical assessments and continued monitoring. However, higher spatial resolution imagery, e.g., Sentinel-2, is necessary to increase the sensitivity of this methodology to fine-scale change. Next steps include applying the method to compare a broader range of sites, mapping areas identified as clusters of change with high spatial resolution imagery and expanding the methods to include the long record of Landsat 5 data.

## Supporting information

S1 Table The results of the linear regression analysis for all watersheds aggregated by acquisition date. (DOCX)

S2 Table The results of the linear regression analysis for micro tidal areas (< 0.8 m) by vegetation type (E2EM1Nd, E2EM1N, E2EM1P, E2EM1Pd). (DOCX)
