# Supplementary material for: Salt marsh monitoring along the mid-Atlantic coast by Google Earth Engine enabled time series
Source: PLoS One. 2020 Feb 28;15(2):e0229605. doi: 10.1371/journal.pone.0229605 (PMC7048292; doi:10.1371/journal.pone.0229605)
Supplement: S1 Table — (DOCX) [file pone.0229605.s001.docx]

Table S1: The results of the linear regression analysis for all watersheds aggregated by acquisition date.

| All Watersheds |  | AGB Trend (1998-2018) |
| --- | --- | --- |
|  | Image Date | 0.423 |
|  | N | 20 |
|  | R^2^ | -0.018 |
|  | F statistic | 0.67 |
