# Supplementary material for: Salt marsh monitoring along the mid-Atlantic coast by Google Earth Engine enabled time series
Source: PLoS One. 2020 Feb 28;15(2):e0229605. doi: 10.1371/journal.pone.0229605 (PMC7048292; doi:10.1371/journal.pone.0229605)
Supplement: S2 Table — (DOCX) [file pone.0229605.s002.docx]

Table S2: The results of the linear regression analysis for micro tidal areas (< 0.8 m) by vegetation type (E2EM1Nd, E2EM1N, E2EM1P, E2EM1Pd)

| E2EM1Nd |  | AGB Trend (1998-2018) |
| --- | --- | --- |
|  | Tidal Range | 2.669 * |
|  | N | 14 |
|  | R^2^ | 0.3373 |
|  | F statistic | 7.126 |
| E2EM1N |  | |
|  | Tidal Range | 0.726 |
|  | N | 30 |
|  | R^2^ | 0.01727 |
|  | F statistic | 0.5272 |
| E2EM1Pd |  | |
|  | Tidal Range | 2.497* |
|  | N | 34 |
|  | R^2^ | 0.155 |
|  | F statistic | 6.237 |
| E2EM1P |  | |
|  | Tidal Range | 0.045 |
|  | N | 44 |
|  | R^2^ | 0.00004678 |
|  | F statistic | 0.002 |

*Significant at 0.05 level
